# Editorial

**Published:** 1839-08-03

**Authors:** 


### THE MEDICAL EXAMINER.

PHILADELPHIA, AUGUST 3, 1839.

The bill for the incorporation of the Medical College of Philadelphia, passed both houses of the Legislature at their last session, and now waits only the signature of the governor, to become a law. This liberal plan, which secures to every physician additional facilities for entering upon the duties of medical instruction, has received the almost unanimous support of the medical profession in this city; and, as far as we know, has scarcely met with opposition from those members of the profession who are apparently interested in opposing it. This is greatly to the honour of those gentlemen, and is in accordance with their true interests; for if the plan should succeed, the established schools will possess great advantages over any newly-formed combination, and will be sustained, instead of being injured, by the examining college.

We do not know what are the exact provisions of the bill; we understand, however, that they are, in substance, those originally proposed by the Medical College. That is, the college, which is a very numerous body, is to appoint a Board of Examiners, who will grant diplomas to those properly qualified. Some restrictions, we understand, were introduced by the Legislature, the object of which was, probably, to require from the student that he should attend at least one course of lectures at Philadelphia.

Our reasons for desiring the passage of this bill, are, that it seems to us well calculated to elevate the standard of medical instruction and increase the diffusion of sound professional knowledge, and that it sanctions a mode of testing the qualifications of physicians, which will undoubtedly find favour with the profession. We fear that if the bill should, by any chance, become inoperative, that some similar plan will be carried into effect, which may be without the sanction of a state Legislature, and will, therefore, carry with it less apparent authority. It is the moral sanction which is desirable, and will tend to keep up the distinction between the educated physician and the mere pretender. The legal barriers against irregular practitioners, are now almost entirely overthrown. It may be better that it is so; at any rate, it is consistent with the progress of the times, and cannot now be remedied. The moral influence of a well-educated physician remains, although his legal privileges are lost; and we believe that the Medical College, if successful, would tend to strengthen this moral authority, by increasing both the facilities for instruction, and the requisitions necessary for a diploma. We feel an additional interest in the new college, because it adds to the facilities offered by Philadelphia as a school of medical instruction, and at the same time tends to sustain the institutions which have already acquired a well-earned reputation.

---

There is a question of medical ethics which, directly or indirectly, interests all members of the profession;—it is, that of consultations by letter. Physicians placed under certain circumstances, are frequently honoured by letters asking for advice in a particular case. Those who receive letters of this kind are either long known in the profession, and have acquired a reputation for skill in the treatment of medical or surgical diseases in general, or they are those who have devoted themselves to the especial study of a particular class of affections.

In either case much time and labour have necessarily been expended, before the reputation which prompts such consultations is acquired; and it is necessary for the advantage of the patient and the physician, that these applications should be placed upon a proper footing. In order to benefit the patient, the actual state of the case should not only be detailed with accuracy, but the hour of its commencement, the previous health and habits of the patient, and his liability to hereditary disease, should be carefully insisted upon. If these circumstances be related in a distinct and accurate manner, the opinion of the consulting physician may be given nearly with the same accuracy as if the patient were actually before him. The only insurmountable disadvantages would be, the impossibility of resorting to the physical means of investigation, and of conducting a continuous plan of treatment.

In such consultations, it is the duty of the physician to consult the just interests of his professional brethren. To do justice to an application of the kind, a physician must carefully read a letter of some length—should give it deliberate consideration, and must reply at such length as to remove all chances of having his meaning imperfectly understood. Now, all this demands the sacrifice of time, which is by no means inconsiderable, especially as it is required from physicians who usually have many arduous duties to perform. It is, therefore, but an act of justice that the patient, for whose ultimate benefit the advice is given, should understand that the labour and responsibility of the consulting physician are greater than in cases in which he is actually present. The compensation which he receives ought not, of course, to be paid by the attending physician; but the patient should be informed of the circumstances, and instructed to send an adequate fee at the time the application is made.

We make these remarks because we have ourselves felt their necessity, and still more because some of our professional brethren have complained of the rather heavy tasks which are sometimes required of them. The better understanding of the matter would conduce to the interests of all parties, and secure a just remuneration for professional labour, which is not very highly paid under any circumstances.

